# Interspecific secondary plasmodesmata at the parasitic interface

**DOI:** 10.1093/pcp/pcaf143

**Published:** 2025-11-03

**Authors:** Koh Aoki, Ayako Tsushima

**Affiliations:** Graduate School of Agriculture, Osaka Metropolitan University, 1-1 Gakuen-Cho, Naka-Ku, Sakai 599-8531, Japan; Graduate School of Agriculture, Osaka Metropolitan University, 1-1 Gakuen-Cho, Naka-Ku, Sakai 599-8531, Japan

**Keywords:** *Cuscuta*, obligate parasite, parasitic interface, parasitic plants, secondary plasmodesmata

## Abstract

Obligate parasitic plants often establish symplastic connections with their hosts. This symplastic continuity is mediated by plasma membrane-lined channels referred to as interspecific secondary plasmodesmata, which develop at the interface between the parasite and its host. However, the molecular mechanisms underlying the formation of these interspecific secondary plasmodesmata remain unclear. In this mini review, we summarize current knowledge of plasmodesmata biogenesis at diverse cellular boundaries with distinct developmental origins, including those at graft junctions and between mesophyll and bundle sheath cells. Based on the literature review, we hypothesize that the formation of interspecific secondary plasmodesmata involves three key events: cell wall thinning, membrane rearrangement, and metabolic regulation. Finally, we discuss future research directions to elucidate the molecular basis of interspecific secondary plasmodesmata formation.

## Introduction

Within the angiosperms, parasitic plants are known to have evolved independently at least 12 times (Twyford 2018). These plants obtain some or all of their nutrients from living host plants. Parasitic plants can be categorized into two types, namely facultative parasites, which can complete their life cycle without a host, and obligate parasites, which rely on a host for survival and reproduction. Despite this difference in host dependency, parasitic plants generally form a specialized multicellular structure called a haustorium (Yoshida et al. 2016). The haustorium develops at the site of host contact, penetrates host tissues, and establishes a vascular connection that enables the transfer of nutrients and other molecules.

The haustorium is essential for establishing parasitism, but its mere formation is not always sufficient for nutrient absorption. Parasitic plants must also establish functional vascular continuity with their hosts. Obligate parasitic plants, which exhibit greatly reduced photosynthetic activity, develop both phloem and xylem conducting cells within their haustoria. These cells form direct connections with the host’s phloem and xylem elements, allowing the parasitic plants to obtain essential nutrients, such as photosynthates and amino acids (Birschwilks et al. 2006, Kokla and Melnyk 2018). Facultative parasitic plants, which retain photosynthetic competence, develop xylem vessels within their haustoria that connect to the xylem vessels of the host (Spallek et al. 2017). The formation of a direct phloem connection between haustoria and hosts varies among species. For example, *Phtheirospermum japonicum*, a facultative root parasite, establishes a direct connection between its sieve elements and those of the host (Masumoto et al. 2021), whereas *Psittacanthus schiedeanus*, a facultative mistletoe, does not form such connections (Cocoletzi et al. 2016). Unlike xylem, phloem conducting elements comprise living cells known as sieve elements (Lucas et al. 2013). Consequently, substances that are transported through phloem connections must move intercellularly. In plants, direct cell-to-cell transport occurs via the symplastic pathway, which is mediated by plasmodesmata. Plasmodesmata are plasma membrane (PM)-lined channels that interconnect almost all neighboring plant cells (Epel 1994, Lucas 1995, Zambryski 1995). They have also been identified between cells of different plant species, including parasitic plants and their hosts (Vaughn 2003, Birschwilks et al. 2006, Shimizu et al. 2018). Nonetheless, the molecular mechanisms responsible for plasmodesmata formation at the parasitic interface remain poorly understood.

In this mini review, we summarize the literature on plasmodesmata biogenesis at cellular boundaries formed between cells of distinct developmental origins, including those at graft junctions and at the boundary between mesophyll cells (MCs) and bundle sheath cells (BSCs). Based on the available information, we propose possible mechanisms for the formation of interspecific secondary plasmodesmata. Finally, we discuss research perspectives aimed at elucidating the regulatory mechanisms underlying the establishment of symplastic connections between parasitic plants and their hosts.

## Definition of Primary and Secondary Plasmodesmata

In discussing how interspecific plasmodesmata form, it is necessary to revisit the distinction between primary and secondary plasmodesmata (T.M. Burch-Smith et al. 2011b). Primary plasmodesmata arise specifically during cell division, when endoplasmic reticulum (ER) tubules span the phragmoplast and become incorporated into the developing cell plate (Staehelin and Hepler 1996). As the cell plate matures, cytosolic strands surrounding these ER tubules constrict, giving rise to primary plasmodesmata. These newly formed channels are initially unbranched, evenly distributed across the new cell wall, and may subsequently acquire more complex structures, including branching and the development of a central cavity. In contrast, secondary plasmodesmata form independently of cell division. Their morphology is highly variable, ranging from simple unbranched channels to branched forms resembling mature primary plasmodesmata. Secondary plasmodesmata occur across a wide range of pre-existing cell walls that separate cells of different developmental origins. Examples include regenerating protoplasts (Ehlers and Kollmann 1996), longitudinal walls of rapidly elongating cells (Seagull 1983), graft junctions (Notaguchi et al. 2020), and specialized interfaces between MCs and BSCs in C_4_ plants (Edwards et al. 2004).

## Plasmodesmata at the Parasitic Interface

**Figure 1:**
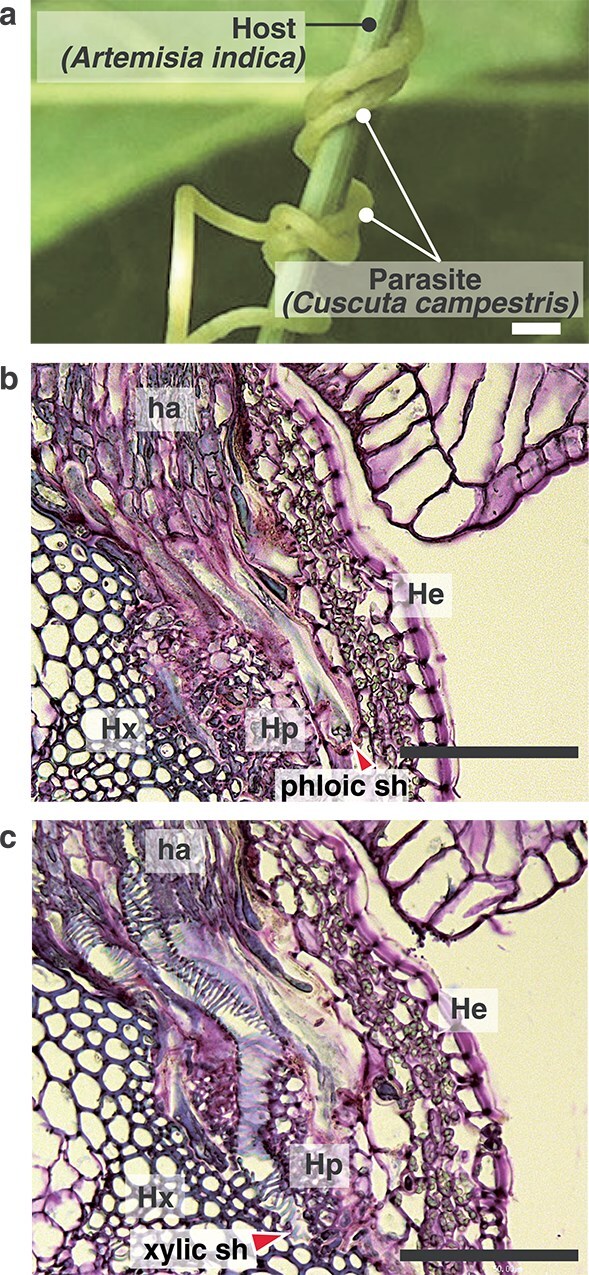
Parasitic interface of the stem obligate parasite *C. campestris*. (a) Appearance of the parasitic interface between *C. campestris* and the stem of *Artemisia indica*. Scale bar, 500 μm. (b, c) Transverse paraffin sections (thickness, 10 μm) of a *C. campestris* haustorium penetrating the stem of *A. thaliana*. Sections were stained with toluidine blue. Scale bars, 50 μm. (b) and (c) Serial sections of the same haustorium at different depths. (b) Focused on a phloic hypha; (c) focused on a xylic searching hypha. Ha: Haustorium, phloic sh: Searching hypha differentiating into a phloem-conducting element, xylic sh: Searching hypha differentiating into a xylem-conducting element, hx: Host xylem, hp: Host phloem, He: Host epidermis. Arrowheads indicate the tips of phloic and xylic hyphae. The phloic sh exhibits an enlarged tip region, where transfer of phloem-localized substances from host sieve elements has been reported (Park et al. 2021). The xylic sh shows characteristic secondary cell wall thickening similar to differentiated tracheary elements, and establishes direct contact with host xylem vessels, although this interaction is not visible in the image.

Plasmodesmata develop at the interface between cells of different plant species at the host–parasite boundary. Fischer et al. (2021) provided a comprehensive summary of studies on symplastic connections between parasitic plants and their hosts, covering both ultrastructural and macromolecular transport aspects. The plasmodesmata involved in these symplastic connections are considered secondary because the cell walls between the parasite and host cells do not develop from cell division. Here we describe an example of the parasitic interface of the stem obligate parasite, *Cuscuta campestris* (Fig. 1a). The interfaces between the elongated cells on the haustorium tip, termed searching hyphae, and the host cell walls are the sites where interspecific plasmodesmata formation occurs. (Fig. 1b and c). Searching hyphae of *C. campestris* can differentiate into either xylem vessels (Fig. 1b) or phloem-conducting elements (Fig. 1c).

Obligate parasitic plants, such as *Cuscuta reflexa* and *Orobanche cumana*, form direct connections between their sieve tubes and those of the host (Haupt et al. 2001; Lee 2009; Krupp et al. 2019), developing sieve pores at the interfacial cell wall (Krupp et al. 2019). It has been established that the sieve pores originate from preexisting plasmodesmata (Kalmbach and Helariutta 2019). The diameter of these plasmodesmata increases due to local pectin degradation by the enzyme pectate lyase (PLL12) (Kalmbach et al. 2023). Consequently, the presence of interspecific sieve pores indicates that interspecific plasmodesmata are formed prior to their development.

Plasmodesmata have also been observed at the interface between haustorial tips and host parenchyma cells in addition to interspecific sieve pores (Vaughn 2003, 2006, Birschwilks et al. 2006). Vaughn (2003) described interspecific plasmodesmata in the cell walls of searching hyphae in the stem obligate parasite *C. pentagona*. That study also reported that plasmodesmata occur more frequently near the tips of searching hyphae, consistent with a previous finding in *C. reflexa* in which plasmodesmata were confirmed at the searching hyphae cell wall using the GFP-fused *Tobacco Mosaic Virus* movement protein (Birschwilks et al. 2006), which localizes to both simple and branched plasmodesmata (Fitzgibbon et al. 2010).

These observations collectively indicate the presence of interspecific secondary plasmodesmata at the parasitic interface. However, the mechanisms underlying the formation of these interspecific secondary plasmodesmata remain poorly understood. In the following sections, we explore the potential molecular basis for plasmodesmata formation. This involves a review of processes common to well-studied systems, including cell–cell interactions at graft junctions and the interface between MCs and BSCs in C_4_ plants.

## Cell Wall Thinning

Cell wall thinning is a perquisite for establishing cell-to-cell contact and subsequent plasmodesmata formation. At the graft junction, pre-existing cell walls are locally loosened and thinned upon contact between scion and the stock tissue (Notaguchi et al. 2020). This allows for contact of the opposing PMs and subsequent formation of plasmodesmata strands during cell wall reconstruction. Notaguchi et al. (2020) identified the gene *NbGH9B3*, which encodes the cell wall-degrading enzyme GLYCOSYL HYDROLASE 9B3 in *Nicotiana benthamiana*, and is specifically upregulated during graft union formation in interfamily grafting with *Arabidopsis thaliana* stock. As *NbGH9B3* encodes a β-1,4-glucanase secreted into the apoplast, it likely targets cellulose in the cell wall and contributes to cell wall reorganization. At the graft junction, synchronized cell wall thinning on both the scion and stock sides is proposed to establish secondary plasmodesmata, whereas insufficient coordination in wall thinning is considered to generate half- or outer-wall plasmodesmata (Fischer et al. 2021). However, the signaling molecules that are exchanged between the scion and the stock to mediate synchronized cell wall thinning remain to be identified. Besides the membrane rearrangement, cell wall remodeling facilitates physical contact between cells for plasmodesmata development.

Local thinning of interspecific cell walls has been observed at the parasitic interface between *C. reflexa* and *P. japonicum* with their respective hosts (Lee 2009; Kurotani et al. 2020). Therefore, a similar cell wall reorganization process is expected at the parasitic interface. This point is discussed in greater detail in Section “Hypotheses for the mechanisms of parasitic secondary plasmodesmata formation.”

## Membrane Rearrangement

One of the key processes in plasmodesmata development is the formation of a desmotubule across the cell wall, which requires dynamic reorganization of membranes. This desmotubule consists of a tubular ER strand surrounded by the PM. It has been proposed that initial ER–PM contact is a prerequisite for desmotubule formation. This concept is supported by observations that, on the surface of regenerating protoplasts, the ER comes into close contact with the PM immediately after cell wall removal, and ER tubules with both ends anchored within the same protoplast, referred to as half-plasmodesmata, are frequently observed in the newly regenerated cell walls (Ehlers and Kollmann 1996).

The interaction between the ER and PM during plasmodesmata formation has been further investigated at graft junctions, where scion and stock cells come into contact. In regions with thinner cell walls, the ER and PM are found in close apposition. This observation suggests that ER–PM contact is necessary for desmotubule formation (Chambaud et al. 2022). Plasmodesmata spanning the entire cell wall are typically associated with relatively thin cell walls, whereas hemi-plasmodesmata, or half-plasmodesmata, generally occur in areas where the cell wall is thicker (Chambaud et al. 2022). Chambaud et al. (2022) proposed that desmotubules extend into the cell walls following ER–PM contact. They also noted that the penetration depth of the cell wall-spanning plasmodesmata and hemi-plasmodesmata does not differ significantly, suggesting that desmotubules can extend across the entire cell wall when local thinning occurs, but fail to do so in regions of greater cell wall thickness.

Proteins that tether membranes play an important role in establishing contact between the EM and the PM (Manford et al. 2012). Several families of tethering proteins are associated with plasmodesmata, including reticulons (Knox et al. 2015), synaptotagmins (SYTs; Ishikawa et al. 2017), vesicle-associated membrane protein-associated proteins (Wang et al. 2016), and multiple C2-domain and transmembrane region proteins (MCTPs; Brault et al. 2019). Among these, specific members of the MCTP family, i.e. MCTP3, MCTP4, and MCTP6, play key roles in the formation of plasmodesmata (Li et al. 2024, Pérez-Sancho et al. 2025). In the *mctp3/4/6* triple mutant, loss of ER–PM tethering is observed within the cytoplasmic sleeves of plasmodesmata (Pérez-Sancho et al. 2025). This attachment is mediated by interactions between the MCTPs and the membrane lipid phosphatidylinositol-4-phosphate (Pérez-Sancho et al. 2025). Although these analyses primarily examined the primary plasmodesmata in post-cytokinetic cell walls, Li et al. (2024) demonstrated that the plasmodesmata density is reduced in various root tissues of the mutant (Li et al. 2024). This finding suggests that MCTPs may regulate plasmodesmata biosynthesis. In addition, the study showed that MCTPs stabilize ER strands in nascent plasmodesmata and prevent disruption of cytoplasmic continuity.

Collectively, these results highlight the importance of ER–PM interactions in the formation and structural stabilization of plasmodesmata. While much of this knowledge is derived from studies of primary plasmodesmata, it provides a framework for understanding the formation of interspecific secondary plasmodesmata. This topic will be elaborated in a Section “Hypotheses for the mechanisms of parasitic secondary plasmodesmata formation.”

## Metabolic Regulation

Formation of plasmodesmata in non-dividing cell walls has been extensively studied at the interfaces between MCs and BSCs. Because these cells have distinct ontogenetic origins, the plasmodesmata are classified as secondary plasmodesmata (Edwards et al. 2004). The number of plasmodesmata connecting MCs and BSCs increases under light irradiation (Schreier et al. 2024). When C_4_ plants are treated with inhibitors of photosynthesis, such as 3-(3,4-dichlorophenyl)-1,1-dimethylurea, or inhibitors of chloroplast development, such as norflurazon, the frequency of plasmodesmata at MC–BSC interfaces decreases significantly (Schreier et al. 2024). Interestingly, whereas a metabolizable sucrose rescued this reduction, the nonmetabolizable analog of sucrose, turanose, did not (Schreier et al. 2024). These findings indicate that plasmodesmata formation at the MC–BSC interface is regulated by chloroplast-associated metabolism.

**Figure 2:**
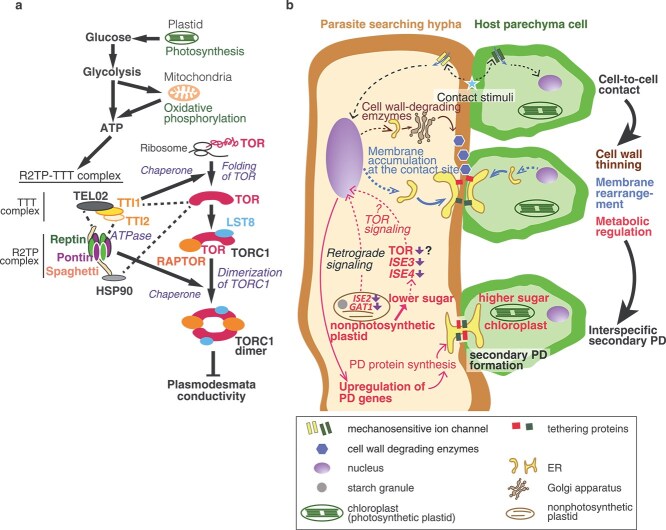
Target of rapamycin (TOR) signaling pathway and hypothetical key events in the formation of the interspecific secondary plasmodesmata. (a) Schematic diagram of target of rapamycin (TOR) signaling. When plastid photosynthetic activity is high, cytosolic glucose levels increase. This rise enhances glycolysis and oxidative phosphorylation, leading to higher ATP production. Elevated ATP levels promote cochaperone activity of the R2TP–TTT complex. The TTT complex assists in TOR protein synthesis, and together the R2TP and TTT complexes cooperate with HSP90 to facilitate TORC1 dimerization, the active form of TORC1. This activation of TORC1 represses plasmodesmata conductivity. Conversely, when TORC1 activity is low, such as during reduced glucose availability of inactive photosynthesis, plasmodesmata conductance increases. Arrows indicate positive regulation; blunt-end arrows indicate negative regulation; dashed lines indicate protein–protein interactions. (b) Hypothetical key events in interspecific secondary plasmodesmata formation, inferred from processes observed at graft junctions, in contacting cells, and at MC–BSC interfaces. When a parasite’s searching hypha comes into contact with a host cell, this interaction is detected by ion channels, although the specific ion channel responsible has not been identified. This contact induces cell wall thinning and membrane reorganization, which may occur interdependently. Interaction between the searching hypha and the host cell activates secretion of cell wall-degrading enzymes into the interspecific wall (indicated in the upper part of the panel). Cell wall thinning brings the plasma membranes into closer apposition, triggering accumulation of ER at the interface (indicated in the middle part of the panel). These events are likely crucial for interspecific secondary plasmodesmata formation, consistent with the requirement of specific cellulases for parasitism in root hemiparasitic plants (Kurotani et al. 2020). Additionally, differences between plastid photosynthetic activity between the parasite searching hyphae and the host cells resemble those at the MC–BSC interface (indicated in the lower part of the panel). On the parasite side, lower photosynthetic activity may downregulate the activity of the TOR signaling pathway and organelle-localized plasmodesmata regulators such as *ISE2* and *GAT1*. Although the relationship between increased plasmodesmata conductivity and plasmodesmata formation is not fully resolved, downregulation of these regulatory factors could upregulate genes encoding plasmodesmata component proteins, as reported in *ise1* and *ise2* mutants (T.M. Burch-Smith et al. 2011a), potentially enhancing secondary plasmodesmata formation. PD: plasmodesmata.

The mechanism underlying the increased frequency of plasmodesmata in response to photosynthetically derived sugars remains unresolved. In this section, we summarize current evidence for sugar-dependent regulation of plasmodesmata conductivity, noting that plasmodesmata frequency and conductivity are not necessarily correlated at the MC–BSC interface (Gao et al. 2022). Brunkard et al. (2020) demonstrated a link between sugar metabolism and plasmodesmata conductivity. The target of rapamycin (TOR) metabolic signaling pathway is well known to coordinate metabolism with nutrient availability in eukaryotes (Fig. 2a). The TOR is a highly conserved serine/threonine kinase found in eukaryotes (Y. Liu et al. 2025). TOR activity increases under nutrient-sufficient conditions and decreases under nutrient limitation (Saxton and Sabatini 2017). The TOR is active when a TOR dimer associates with Regulatory-Associated Protein of TOR and Lethal with SEC13 Protein 8 (LST8) to form TOR complex 1 (TORC1) (Saxton and Sabatini 2017). Activation of TORC1 is promoted by dimerization, a process facilitated by a cochaperone complex consisting of HEAT SHOCK PROTEIN 90 (HSP90) and the R2TP-TTT complex (Fig. 2a). The R2TP subcomplex is composed of Pontin/RuvBL1, Reptin/RuvBL2, Spaghetti/Tah1, and Pih1, whereas the TTT subcomplex is composed of Telo2, Telo2-interacting protein 1, and Telo2-interacting protein 2. Reptin and pontin function together as an ATPase within the R2TP-TTT complex, promoting TORC1 dimerization when ATP levels are high. Under ATP limitation, TORC1 fails to dimerize (Kim et al. 2013; Fig. 2a). When TORC1 is active, it phosphorylates several target proteins, including those in the phosphorylation cascade downstream of Ribosomal Protein eS6 Kinase (S6K) (Saxton and Sabatini 2017). Brunkard et al. (2020) showed that mutants of R2TP complex components, such as the *ise4* mutant, whose causal gene encodes Reptin, and the *spaghetti* mutant, whose causal gene encodes a tetratricopeptide repeat/carboxylate clamp protein with a C-terminal RPAP3-specific domain that recruits HSP90 to the R2TP subcomplex, exhibit increased plasmodesmata transport. In addition, a mutant of TORC1 component, *lst8*, also exhibits increased plasmodesmata transport (Brunkard et al. 2020). These findings indicate that the R2TP and TORC1 complexes regulate plasmodesmata transport, such that transport is enhanced when these complexes are inactive (Fig. 2a). This suggests that reduced metabolic activity upstream of TOR signaling is associated with increased plasmodesmata conductivity. Supporting this model, Brunkard et al. (2020) identified the causal gene of the *ise3* mutant, which encodes mitochondrial SEL1-like repeat-containing protein, a factor required for assembly of complex IV of the oxidative phosphorylation chain. The *ise3* mutant also exhibits increased plasmodesmata transport. Together with the observation of glycolysis inhibition being induced by the non-metabolizable glucose analog 2-deoxy-D-glucose and increased plasmodesmata transport (Brunkard et al. 2020), these findings demonstrate that reduced metabolic activity upstream of the TOR enhances plasmodesmata conductivity (Fig. 2a). Although the specific role of TOR signaling in regulating conductivity between MCs and BSCs remains unresolved (Chen et al. 2024), it is likely that that cellular energy metabolism influences both plasmodesmata formation and conductivity. This interpretation is consistent with the localization of several plasmodesmata regulatory proteins to mitochondria or chloroplasts. *ISE1* encodes a mitochondrial DExH-box ATP-dependent RNA helicase (Stonebloom et al. 2009), whereas *ISE2* encodes a chloroplastic DExH-box ATP-dependent RNA helicase (Kobayashi et al. 2007, T.M. Burch-Smith et al. 2011a). Additionally, *GFP ARRESTED TRAFFICKING 1* (*GAT1*) encodes a chloroplastic thioredoxin (Benitez-Alfonso et al. 2009). Collectively, these studies suggest a potential feedback between cellular metabolic status and plasmodesmata formation.

## Transcriptional Regulation of Plasmodesmata Component Proteins

Formation of secondary plasmodesmata prompted us to investigate whether this process is associated with the expression of plasmodesmata-related genes. In *A. thaliana*, plasmodesmata contain more than 60 proteins (Kirk et al. 2022). The functions of these proteins have been reviewed comprehensively elsewhere (e.g. Zanini and Burch-Smith 2024). During graft union formation in interfamily grafting between *N. benthamiana* and *P. japonicum*, more than 25 plasmodesmata-related genes are upregulated (Kurotani and Notaguchi 2021). In the C_4_ monocot, *Setaria viridis*, the density of plasmodesmata clusters, or pit fields, correlates with differential expression of plasmodesmata- and photosynthesis-related genes (Tsang et al. 2024). In the loss-of-function mutant, *ise1* (Stonebloom et al. 2009), *BETA-1,3-GLUCANASE,* and *PLASMODESMATA CALLOSE-BINDING PROTEIN 3* (*PDCB3*) were downregulated, while *GLUCANSYNTHASE-LIKE 8* (*GSL8*) and *PLASMODESMATA-LOCATED PROTEIN 8* (*PDLP8*) were upregulated. In contrast, in the loss-of-function mutant, *ise2*, *BETA-1,3-GLUCANASE*, *GSL8*, and *PDCB3* were downregulated, while *CASEIN KINASE 1*, *PDLP5*, *PDLP6*, and *REVERSIBLY GLYCOSYLATED PROTEIN 3* were upregulated (Burch-Smith and Zambryski 2010). These examples collectively suggest that interspecific secondary plasmodesmata formation is accompanied by transcriptional regulation of plasmodesmata-related genes.

## Hypotheses for the Mechanisms of Parasitic Secondary Plasmodesmata Formation

Integrating current knowledge of plasmodesmata development, we discuss potential mechanisms underlying interspecific secondary plasmodesmata formation (Fig. 2b).

At the parasitic interface, secondary plasmodesmata formation likely begins when haustoria contact host cells. Local cell wall thinning is thought to be initiated upon contact, enabling membrane–membrane apposition. Profiling of cell wall components, including polysaccharides and glycoproteins, in diverse parasitic plants has demonstrated spatiotemporal accumulation patterns during haustorium development (Johnsen et al. 2015). Consistent with this, the expression of genes encoding cell wall-related enzymes shows dynamic changes at the parasitic interface (Bawin et al. 2023, Chen et al. 2024). Interestingly, expression of endo-β-1,4-mannanase genes in *C. campestris* increases in response to purified mono- and polysaccharides and correlates with mannan content in infected host tissues, suggesting that host cell wall-derived signals can induce expression of genes involved in cell wall degradation (Bawin et al. 2023). The importance of cell wall reorganization in parasitization is supported by the observation that *C. campestris* knock-down of *PECTIN METHYLESTERASE INHIBITOR* (*PMEI*), a determinant of pectin degradation, results in fewer haustoria and vascular connections (Jhu et al. 2022). Similarly, knock-down of *P. japonicum* homologs of *NbGH9B3* (*PjGH9B3*) reduces the success rate of xylem connection, indicating its involvement in cell–cell attachment at the parasitic interface. However, the formation of interspecific secondary plasmodesmata has not yet been demonstrated in this facultative parasite (Kurotani et al. 2020). Homologs of *GH9B3*, *GH9B7*, *GH9B11*, and *GH9B12* in *C. campestris* show differential expression between susceptible and resistant tomato lines (Edema et al. 2024), suggesting their involvement in cell wall reorganization during infection (Fig. 2b). While cell wall-modifying enzymes likely promote parasitization, it remains unsolved whether these enzymes play specific roles in forming interspecific secondary plasmodesmata. Investigating the relationship between cell wall modification and interspecific secondary plasmodesmata should therefore be a priority for future studies.

The thinning of cell walls enhances membrane rearrangement at the contact site. Cell–cell contact generates mechanical stimuli and likely triggers interactions between the ER and PM. On the parasite side of the interfacial cell wall, accumulation and attachment of endomembranes to the PM are frequently observed in the vicinity of interspecific secondary plasmodesmata (Vaughn 2003, 2006). In hyphae destined to differentiate into phloem, accumulation of smooth ER at the interfacial PM has been reported (Vaughn 2006, Krupp et al. 2019). Cell-to-cell contact likely induces membrane deformation, which is generally perceived by mechanosensitive ion channels on the membrane (Hamilton et al. 2015). Although the role of ion channels in interspecific plasmodesmata formation remains unresolved, recent work demonstrated that Ca^2+^ channels encoded by *MID1-COMPLEMENTING ACTIVITY 1* are essential for initiating haustorium development in the stem parasite *C. campestris* (Park et al. 2025). This finding suggests that the haustorium may employ a mechanosensory system that enables recognition of host cells through physical contact. Subsequently, accumulated ER would be anchored to the PM by ER-PM tethering proteins, such as MCTPs and SYTs. The PI4P phosphatase SAC7 may modulate ER-PM contacts by reducing PI4P levels, thereby sustaining the high conductivity of newly formed plasmodesmata (Pérez-Sancho et al. 2025).

The potential metabolic regulation of interspecific plasmodesmata formation has barely been investigated. To address this question, we first examine parallels between the host–parasite interface and the MC–BSC interface. First, at the host–parasite interface, photosynthetic activity on the obligate parasite side is markedly lower than on the host side (Machado and Zetsche 1990), primarily due to the loss of photosynthesis-related genes in obligate parasitic plants (Krause 2011). The MC–BSC interface also exhibits a steep gradient in photosynthetic capacity, with chloroplasts in BSCs having little or no PSII activity, whereas those in MCs possess both active PSII and PSI (Chapman et al. 1980). Second, plastids of searching hyphae contain large starch granules, suggesting that sugar metabolism is active in these cells, which act as a dominant sinks (Birschwilks et al. 2006). At the MC–BSC interface, MCs are exporters of photoassimilates and are regarded as source cells (Daloso et al. 2023), whereas BSCs are regarded as sink cells. Third, plasmodesmata-related genes, including *PDLP3/5/6/8*, *PDCB5*, *BETA-1,3-GLUCANASE*, and *MCTP1/4/12*, are upregulated in haustoria (Kaga et al. 2020, Jhu et al. 2022). In the MC–BSC complex, plasmodesmata-related genes, such as *PDLP8*, *PDCB3/4*, and *BETA-1,3-GLUCANASE* are preferentially expressed in BSCs, indicating that plasmodesmata development at the MC–BSC interface may be governed by BSC-specific regulatory networks (Chang et al. 2012). These similarities led us to hypothesize that sugar metabolism regulates the expression of plasmodesmata-related genes during the formation of interspecific secondary plasmodesmata. Based on evidence from searching hyphae and BSCs, we hypothesize that reduced light-reaction activity in plastids and the sink status of these cells decrease intracellular glucose levels. This reduction may deactivate the TOR pathway. Consequently, decreased TOR activity could increase plasmodesmata conductivity (Brunkard et al. 2020), potentially stimulating *de novo* plasmodesmata formation (Fig. 2b). A gene regulatory network involving organellar regulators of plasmodesmata conductivity, such as *ISE1*, *ISE2*, and *GAT1*, is also likely to contribute to the transcriptional regulation of plasmodesmata-related genes during the formation of new secondary plasmodesmata (Benitez-Alfonso et al. 2009, T.M. Burch-Smith et al. 2011a).

Taken together, at the parasitic interface, contact between searching hyphae cells and host cells induces cell wall thinning through the upregulation of genes associated with cell wall degradation and remodeling. This process enhances membrane rearrangement, promoting ER–PM contact and formation of desmotubule precursors. In addition, differences in metabolic status arising from variation in photosynthetic activity trigger the upregulation of plasmodesmata-related genes in sink-side cells. This regulation is likely mediated by the TOR pathway, which responds to photosynthate availability (Fig. 2b).

## Open Questions and Future Perspectives

To refine this hypothesis, several questions remain. First, what is the activity state of the TOR pathway in searching hyphae? We assume TOR activity is low due to the limited photosynthetic capacity of these cells. Furthermore, starch accumulation in plastids may reduce soluble sugar concentrations. However, TOR kinase activity has not yet been directly assessed in this context. Second, how does the TOR pathway regulate the expression of plasmodesmata-related genes? *ISE1* and *ISE2* are candidates in this regulatory network, as several plasmodesmata-related genes are upregulated in the loss-of-function lines (T.M. Burch-Smith et al. 2011a). Identifying transcriptional regulators of these genes, which are likely controlled downstream of the TOR pathway, remains a major challenge. Third, what is the role of cells on the source side? Our hypothesis suggests that host cells at the source side of the parasitic interface are less likely to increase plasmodesmata conductivity or induce *de novo* plasmodesmata formation. This interpretation is consistent with observations at the MC–BSC interface, where “sphincter” structures are frequently observed on the MC side of plasmodesmata, reducing conductivity (Gao et al. 2022). Nonetheless, the regulatory mechanisms governing plasmodesmata-related gene expression in source-side cells require further clarification.

How can we answer these questions? In our opinion, improving the spatial resolution of molecular analyses will be essential. Spatial or single-nucleus transcriptomic platforms are promising for uncovering tissue- or cell type-specific transcriptional changes during secondary plasmodesmata development (Fu et al. 2023, Guo et al. 2025). More precise evaluation of interspecific plasmodesmata morphology can be achieved by three-dimensional electron microscopy reconstruction (Zhao et al. 2024). In particular, serial block face scanning electron microscopy has considerable potential for visualizing plasmodesmata ultrastructure at the parasitic interface (Li et al. 2024). Metabolomics at high spatial resolution remains challenging; however, recent mass spectrometry imaging (MSI) technologies, such as matrix-assisted laser desorption ionization-MSI, have successfully achieved cellular resolution (P. Liu et al. 2025a). These advanced MSI technologies could provide crucial insights into metabolites involved in plasmodesmata formation. In addition to high-resolution approaches, robust genetic modification tools will be indispensable. Host-induced gene silencing has been widely used to functionally characterize genes in parasitic plants, given the difficulty of direct transformation (Jhu et al. 2022, Park et al. 2025). Recently, *Agrobacterium*- and *Rhizobium*-mediated stable transformation methods for *C. campestris* have been established (Adhikari et al. 2025, Alles et al. 2025). These methodologies should overcome longstanding barriers in parasitic plant biology and accelerate the functional evaluation of candidate genes identified through high-resolution omics analyses.

## Conclusion

From current knowledge of plasmodesmata formation, we identify three critical processes: cell wall thinning, membrane rearrangement, and metabolic regulation. We hypothesize that these processes are equally important for the establishment of interspecific secondary plasmodesmata. In the future, the integration of high-resolution technologies and advanced genetic approaches will help to identify key factors from both interacting partners and thereby facilitate the clarification of the mechanisms underlying interspecific secondary plasmodesmata formation.

## Data Availability

No new datasets were generated or analyzed in this study.
